# Methylomes as key features for predicting recombination in some plant species

**DOI:** 10.1007/s11103-023-01396-8

**Published:** 2024-03-08

**Authors:** Mauricio Peñuela, Jorge Finke, Camilo Rocha

**Affiliations:** iÓMICAS, Facultad de Ingeniería y Ciencias, Pontificia Universidad Javeriana, 760031 Cali, Colombia

**Keywords:** CHH methylation, Extra Trees, Machine learning, Model plants, Plant breeding

## Abstract

Knowing how chromosome recombination works is essential for plant breeding. It enables the design of crosses between different varieties to combine desirable traits and create new ones. This is because the meiotic crossovers between homologous chromatids are not purely random, and various strategies have been developed to describe and predict such exchange events. Recent studies have used methylation data to predict chromosomal recombination in rice using machine learning models. This approach proved successful due to the presence of a positive correlation between the CHH context cytosine methylation and recombination rates in rice chromosomes. This paper assesses the question if methylation can be used to predict recombination in four plant species: Arabidopsis, maize, sorghum, and tomato. The results indicate a positive association between CHH context methylation and recombination rates in certain plant species, with varying degrees of strength in their relationships. The CG and CHG methylation contexts show negative correlation with recombination. Methylation data was key effectively in predicting recombination in sorghum and tomato, with a mean determination coefficient of 0.65 ± 0.11 and 0.76 ± 0.05, respectively. In addition, the mean correlation values between predicted and experimental recombination rates were 0.83 ± 0.06 for sorghum and 0.90 ± 0.05 for tomato, confirming the significance of methylomes in both monocotyledonous and dicotyledonous species. The predictions for Arabidopsis and maize were not as accurate, likely due to the comparatively weaker relationships between methylation contexts and recombination, in contrast to sorghum and tomato, where stronger associations were observed. To enhance the accuracy of predictions, further evaluations using data sets closely related to each other might prove beneficial. In general, this methylome-based method holds great potential as a reliable strategy for predicting recombination rates in various plant species, offering valuable insights to breeders in their quest to develop novel and improved varieties.

## Key message

Plant species methylation data can be used to predict recombination rates using machine learning regression models; high model performance values on sorghum and tomato data are presented in this paper.

## Introduction

Meiotic recombination is a fundamental process that drives genetic diversity by generating new combinations of existing genetic variation in sexually reproducing organisms (Boideau et al. 2022; Choi 2017). Through the independent segregation of chromosomes and reshuffling of genetic material, meiosis generates novel allelic combinations in offspring (Casale et al. 2022). Recombination not only impacts meiotic outcomes, but also has far-reaching effects on evolutionary and population genomics (Adrion et al. 2020). Specifically, it results in the mixing of alleles between homologous parental chromosomes (Fayos et al. 2022). Although meiotic recombination occurs throughout the genome, crossovers are not evenly distributed and often happen on hotspots in chromosome arms, with a suppression in pericentromeric regions (Choi and Henderson 2015; Colomé-Tatché et al. 2012). In plants, high rates of recombination have been observed in gene-rich euchromatin and low rates in repeat-rich heterochromatin. However, the regulation of meiotic crossover hotspots remains largely unknown (Melamed-Bessudo and Levy 2012; Choi et al. 2013; Choi and Henderson 2015).

Understanding the rates of recombination is crucial for plant breeding. This allows breeders to identify the best varieties for crosses, leading to better offspring development (Brandariz and Bernardo 2019). High recombination rates promote the merging of desirable alleles into one haplotype, but the success of the mix depends on the susceptibility of the chromosomal regions that contain the favorable genes. Hence, knowledge of recombination locations is essential for improving variety development (Brandariz and Bernardo 2019; Casale et al. 2022; Fayos et al. 2022).

Several studies have explored the prediction of chromosomal recombination in multiple species using machine learning methods. Liu et al. (2016) utilized these techniques to construct a predictor for yeast recombination hot/cold spots, while Demirci et al. (2018) applied them to predict recombination in crops such as Arabidopsis, maize, tomato, and rice. Adrion et al. (2020) also employed recurrent neural networks to estimate recombination in *Drosophila melanogaster* populations. Furthermore, Casale et al. (2022) assessed the capability of a genomic prediction approach to reproduce the variation in recombination rate in cultivated barley. Despite the different strategies and features employed by these studies, they all achieved favorable results. However, it is worth noting that these approaches were developed to predict species-level recombination using population data to generate generalized recombination landscapes, rather than predicting recombination in specific varieties that are typically utilized in breeding programs.

Recently, the authors of the present work developed a new approach for predicting recombination rates in two commercial rice varieties using methylomes (Peñuela et al. 2022). This study evaluated the relationship between recombination rates and methylated cytosines in CG, CHG, and CHH contexts (where H is one of C, T, or A) across the twelve rice chromosomes. In that work, CHH methylation was found to have a positive correlation with recombination across chromosomes and was used as a distinct feature to train an Extra Trees model for prediction purposes. This opened up new avenues for using plant methylomes to predict chromosomal recombination. It also may be of value to breeders as it allows for predictions to be made on specific data sets of target varieties, rather than relying on generalized recombination landscapes.

The relationship between DNA methylation and chromosomal recombination has been widely documented, but its precise mechanisms of action remain unclear (Tock and Henderson 2018; Taagen et al. 2020; Fayos et al. 2022; Lloyd 2022). DNA methylation plays a critical role in plant development and serves as a stable mark passed down from one generation to the next, primarily regulated and maintained through DNA replication and cell division by DNA methyltransferases (Law and Jacobsen 2010; Bräutigam and Cronk 2018; Gallo-Franco et al. 2020). DNA methylation is also associated with gene expression regulation, chromosome interactions, and transposon silencing (Zhang et al. 2018). Investigating the natural variations in the epigenome will shed light on the orchestration of gene regulation in plants and the potential for manipulation in the future (Lloyd and Lister 2022).

The ability to predict variations in recombination rates in chromosomes is essential for boosting crop improvement and methylomes hold potential for this purpose. To investigate whether the methylation patterns found in rice and their correlation with recombination can be applied to other plant species, the methodology introduced by Peñuela et al. (2022) was tested on Arabidopsis, maize, sorghum, and tomato, which are model and crop plants. The results indicated that CG and CHG methylation contexts have negative correlation with recombination, while CHH correlated positively in all four tested species. Machine learning models were trained using methylation data to predict recombination rates for each one of the species, with particularly good results obtained for the sorghum and tomato datasets, validating this methodology for both monocotyledonous and dicotyledonous plants.

## Materials and methods

### Data access

Four plant species were evaluated in this study; Arabidopsis, maize, sorghum, and tomato, representing one model plant and three cultivated plants. The raw data for the experiments of bisulfite sequencing were downloaded from GenBank with run accessions SRR9166060, SRR8786631, SRR3286309, and SRR503393, respectively. Recombination rates for each plant species were estimated from the genotype data extracted from the supplementary material of their corresponding articles, namely, Arabidopsis (Singer et al. 2006), maize (Kianian et al. 2018), sorghum (Kimball et al. 2019), and tomato (Gonda et al. 2019).

## Methylation extraction

Illumina bisulfite reads were checked for quality control for each species in FastQC (https://www.bioinformatics.babraham.ac.uk/projects/fastqc/ (accessed on 5 September 2022)). For maize, adaptor remnants were removed using Trimmomatic (Bolger et al. 2014) (http://www.usadellab.org/cms/?page=trimmomatic (accessed September 5, 2022)). The bisulfite reads were aligned with the reference genomes and methylation calls were done using Bismark (Krueger and Andrews 2011) with the default parameters (https://www.bioinformatics.babraham.ac.uk/projects/bismark/ (accessed September 5, 2022). The reference genomes used were TAIR10.1 for Arabidopsis, Zm-B73-REFERENCE-NAM-5.0 for maize, NCBIv3 for sorghum, and SL3.1 for tomato, all of them available in GenBank. Methylated cytosines in the CG, CHG, and CHH contexts were extracted from the Bismark outputs using the Methylkit package in R (Akalin et al. 2012) (https://www.bioconductor.org/packages/release/bioc/html/methylKit.html (accessed September 10, 2022)). Finally, cytosines with methylation levels greater than 75% were retained and counts of these cytosines were performed in windows of 100 kb on each chromosome using Python.

## Recombination rates

For Arabidopsis and tomato, genotype data for each chromosome consisted of a matrix of genetic markers (arranged by sequence position) versus individuals. An entry was encoded as A or B depending on the parental origin of the corresponding sequence. For all chromosomes, new rows with positions every 100 kb were imputed using the nearest marker information. These new rows were used to estimate genetic recombination maps with MapDisto v2 (Heffelfinger et al. 2017) (http://mapdisto.free.fr/ (accessed 10 September 2022)), using the Kosambi mapping function to convert recombination fractions into centimorgans (cM). For maize and sorghum, the genetic map was provided by Kianian et al. (2018) and Kimball et al. (2019), where new rows were imputed every 100 kb with the genetic position in cM using the information of the closest marker. In the case of maize, the female genetic map was used in this study. For all species, these new imputed rows were used to build windows of 100 kb and recombination rates on chromosomes were estimated by subtracting the genetic position of the next window from the current one.

## Machine learning modeling

For each species, a consensus table was made with counts of methylated cytosines in CG, CHG, and CHH contexts, and recombination rates. Exponential smoothing with α = 0.1 was applied to the recombination and methylation data to remove noise associated with the abrupt change in adjacent windows. The smoothed counts of cytosines for all contexts were first correlated with recombination rates and then used as features to train machine-learning models to predict smoothed recombination rates. For each one of the species, the Auto-Sklearn package (Hutter et al. 2019) (https://automl.github.io/auto-sklearn/master/ (accessed 31 October 2022)) was used to identify the regression models with the best prediction performance and their optimal parameters. After choosing the models, an evaluation of the contribution was made by means of Shap values for all the features using the Shap package (https://shap.readthedocs.io/en/latest/index.html (accessed 31 October 2022)). The models were then used to predict the recombination of one chromosome in isolation, trained with information from all remaining chromosomes. This procedure was also developed consecutively for the remaining chromosomes. The value of Pearson’s correlation *r*, the coefficient of determination *R*^2^, and the mean square error *MSE* of each evaluation were estimated to measure the performance of the models. All procedures were developed using Python.

## Results and discussion

**Fig. 1:**
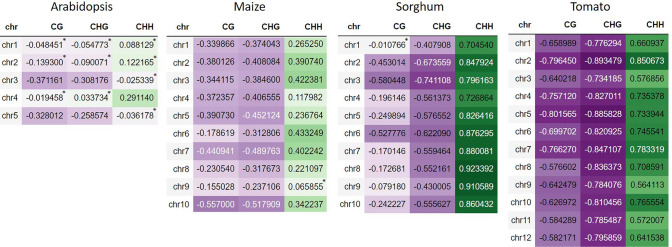
Correlations between recombination rates and the count of methylated cytosines in the contexts CG, CHG, and CHH for chromosomes of *Arabidopsis*, maize, sorghum, and tomato. The purple color represents negative values and the green color represents positive values. The higher the correlation value, the higher the color intensity. Non-significant values are marked with a star

In all cases, the CG methylation context has the highest amount of methylated cytosines, followed by the CHG context, with the CHH context having the lowest amount of methylated cytosines, which is in agreement with the results obtained by Vafadarshamasbi et al. (2022) and with our previous results in rice (Peñuela et al. 2022). Methylation in Arabidopsis, maize, sorghum, and tomato show a positive correlation in CHH context with the recombination rates, whereas methylations in CG and CHG contexts are negatively correlated (Fig. 1). Nevertheless, the values of these correlations are different for each species. In Arabidopsis, for example, positive and negative trends approach zero (between − 0.37 and 0.29). Meanwhile, in maize, correlation trends are somewhat higher (among − 0.55 and 0.43); it is more evident that methylation in the CHH context is positively correlated with recombination. In turn, this presents a higher negative correlation of the CG and CHG contexts. In sorghum, the positive and negative trends are more intense (between − 0.74 and 0.92). In tomato, the highest values are observed when compared to the other three plant species (between − 0.89 and 0.85). The methylation patterns found here are in agreement with the results obtained by Gouil and Baulcombe (2016), which report different methylation landscapes for Arabidopsis, maize, and tomato, depending on the methylation context.

**Fig. 2:**
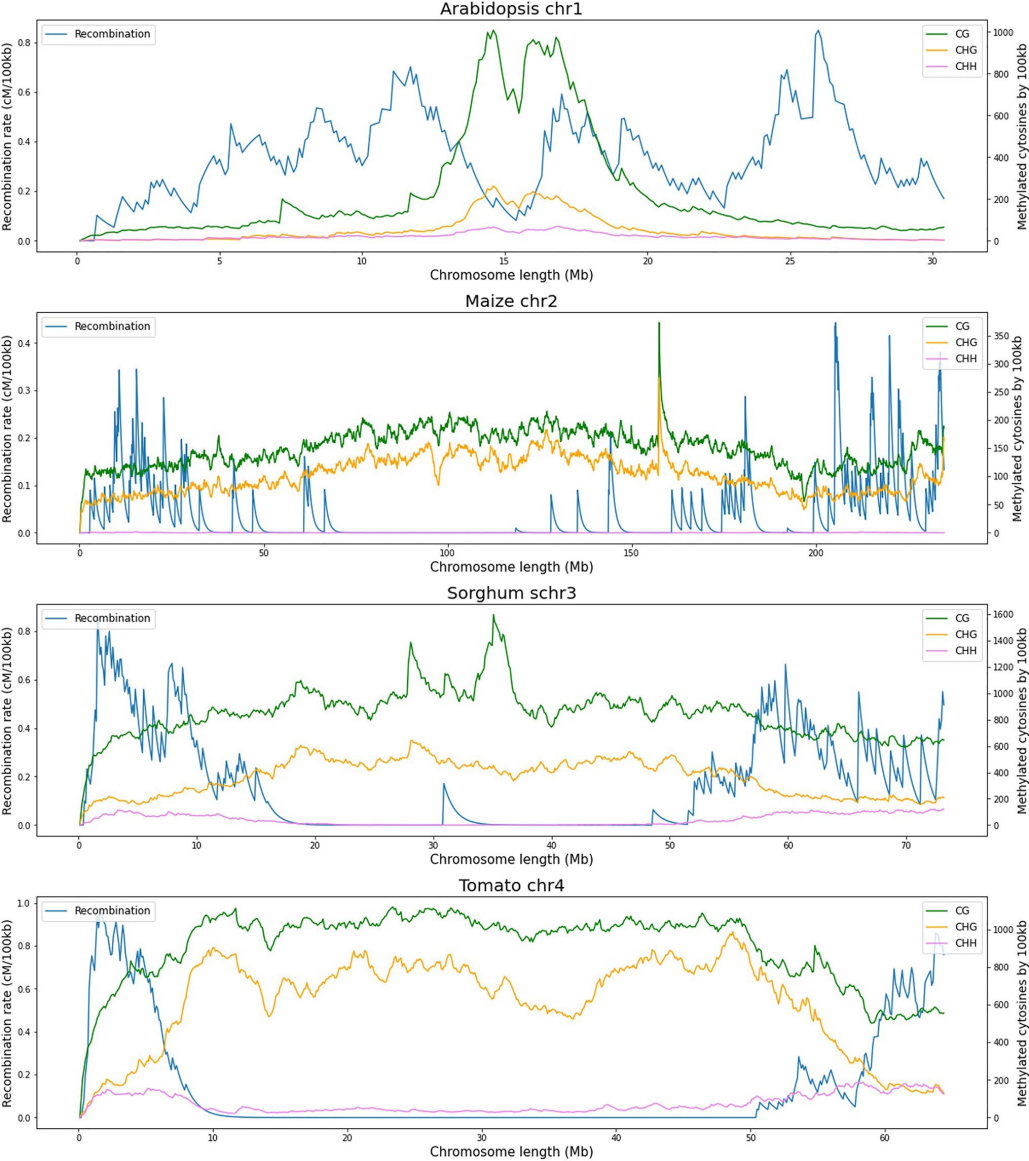
The figure illustrates recombination rates (cM/100 kb) represented in blue, along with counts of methylated cytosines (≥ 75% methylation) per 100 kb window obtained using bismarck and Methylkit. These measurements are categorized into CG (green), CHG (yellow), and CHH (pink) contexts, and pertain to Chromosome 1 of Arabidopsis, Chromosome 2 of maize, Chromosome 3 of sorghum, and Chromosome 4 of tomato

The recombination rates in Arabidopsis chromosomes exhibit a gradual increase from the telomeres towards the pericentromeric regions, where they reach their peak frequencies. However, these rates sharply decline in the centromeric regions (Fig. 2). The same increase from telomere to centromere and suppression of crossovers at the centromere is also reported by Choi et al. (2013) and Rowan et al. (2019). Regarding the number of methylated cytosines in the CG and CHG contexts, they show the opposite path, with the centromeric region exhibiting the highest counts. In fact, there are negative associations for chromosomes 3 and 5 (Fig. 1).

In the case of maize, for example, the highest recombination rates are located at the ends of the chromosome arms, followed by an extended valley where recombination rates are very low, which is a trend in large and complex genomes such as those of grasses (Kianian et al. 2018). It is important to note that maize chromosomes are between five and ten times larger than that of Arabidopsis*.* This is because maize has undergone tetraploidy expansion events and a transposon ‘bloom’ (Horton et al. 2012). However, it is possible to observe a pattern similar to Arabidopsis in which the highest counts of methylated cytosines in the CG and CHG context are found in the center of the chromosome and the lowest ones at the ends, just where the recombination peaks are located.

In maize, recombination predominantly takes place in gene-rich regions while being infrequent in transposon-rich regions, which comprise the majority of the genome (Kianian et al. 2018). Moreover, there have been reports of crossovers occurring at ATG initiation codon positions of genes (Li et al. 2015). These gene regions are unmethylated, whereas transposon regions are highly methylated in CG and CHG contexts (He and Dooner 2009). For maize, (Rodgers-Melnick et al. 2015) found a strong negative relationship between CG and CHG methylation with respect to crossover density in chromosome arms, similar to that reported here. In this study, the same opposite trends between methylation and recombination are clear in the sorghum and tomato chromosomes, which have chromosomes about twice the size of Arabidopsis. For these two species, the negative correlation values shown in Fig. 1 are higher, and the opposite behavior between recombination rates and methylation in CG and CHG contexts is completely evident. As such, methylation plays a crucial role in regulating chromosomal recombination (Tock and Henderson 2018; Taagen et al. 2020; Fayos et al. 2022; Lloyd 2022). For example (Choi et al. 2013), show that gene transcriptional start sites (TSS) and transcriptional terminal sites (TTS), which are in crossover hotspots, presented low methylation compared to non-recombinant sites. In addition, they show that TSSs have a high abundance of histones H2A.Z, H3K4me3, and regions with low nucleosome density (LND). These features increase with recombination rates and show an opposite trend with respect to methylation.

**Fig. 3:**
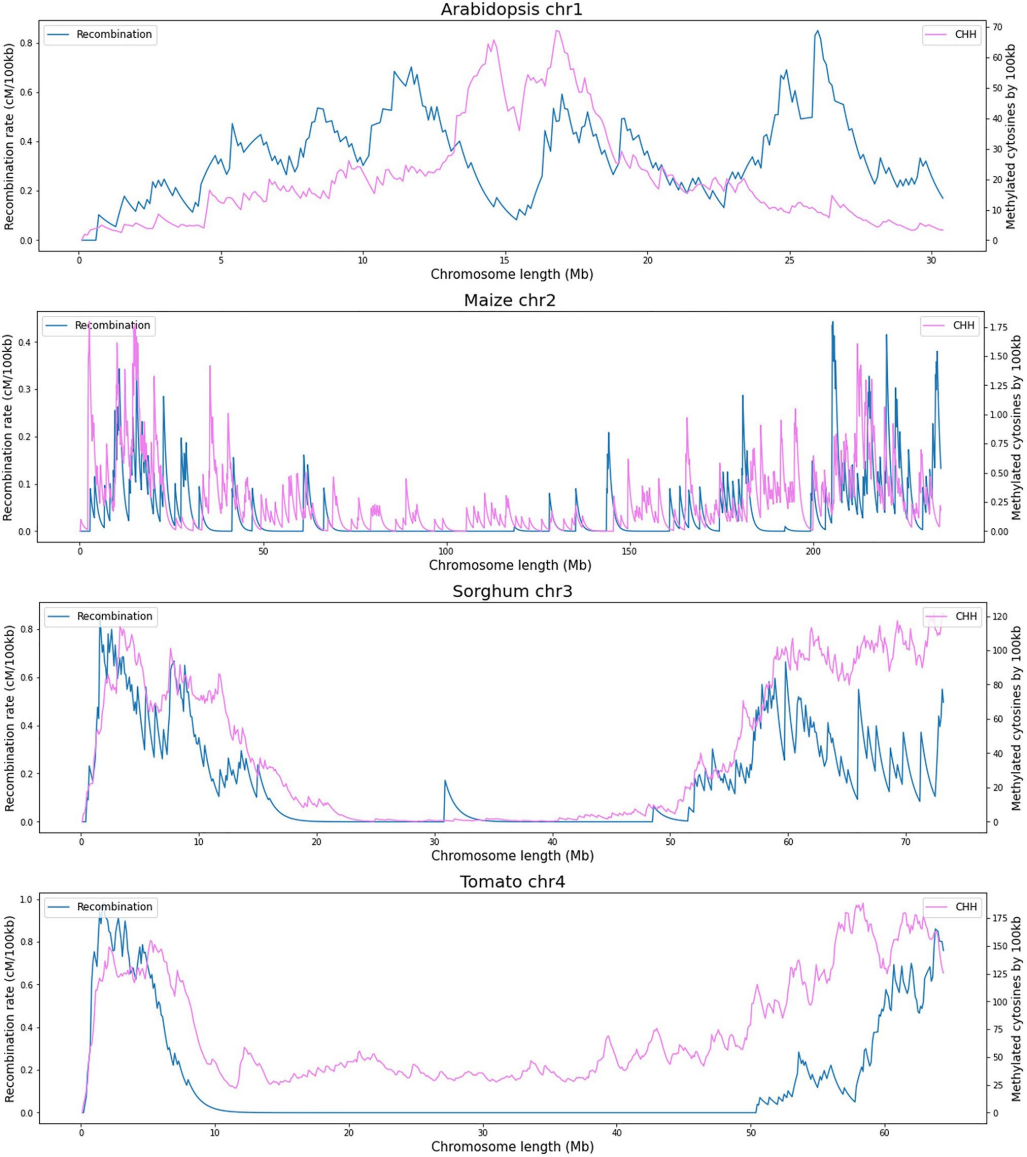
In this figure, recombination rates (cM/100 kb) are depicted in blue, while counts of methylated cytosines within the CHH context (≥ 75% methylation) per 100 kb window, obtained using bismarck and Methylkit, are highlighted in pink. These data correspond to Chromosome 1 of Arabidopsis, Chromosome 2 of maize, Chromosome 3 of sorghum, and Chromosome 4 of tomato

Figure 3 specifically shows the count of methylated cytosines in the CHH contexts, which are low compared to the other contexts and difficult to observe in Fig. 2. Recombination rates and methylated cytosine counts in Arabidopsis increase progressively from the arm ends. However, recombination rates decrease around the centromere region and methylation in the context of CHH increases. For this case, a positive trend between measures is not evident, which can explain why the correlation values are close to zero for Chromosome 1 of Arabidopsis. However, positive values are shown in chromosomes 2 and 4 (Fig. 1). In maize, recombination and methylation rates in CHH contexts show a similar pattern with peaks at the end sections of chromosome arms and a large valley of low values in the middle of the chromosome. This positive relationship between recombination and methylation in the CHH context is supported by the correlation values for most maize chromosomes and have been also reported by Rodgers-Melnick et al. (2015) who used it in their linear regression model. In the case of sorghum and tomato chromosomes, the positive relationship between recombination rates and CHH methylation is strongly supported by high correlation values.

The negative correlation rates for CG and CHG methylation, and the positive correlation rates for CHH methylation with respect to recombination obtained here, are in agreement with the trends reported in maize and rice (Rodgers-Melnick et al. 2015; Peñuela et al. 2022). It supports the hypothesis that methylation in the CHH context plays a key role in chromosomal recombination in plant species. It is not clear what is the biological role of the CHH cytosines. (Stroud et al. 2014) show that the CMT2 protein specifically adds methyl groups to CHH sequences when bound to methylated H3K9. Some studies have reported the role of CHH methylation in fruit size, transposons silencing, seed dormancy, and plant reproductive organs (Daccord et al. 2017; Zakrzewski et al. 2017; Zhang et al. 2018; Wang et al. 2022). In fact, the CHH methylome has been shown to exhibit differences between chromosome arms and the pericentromeric region in plants (Gouil and Baulcombe 2016).

**Fig. 4:**
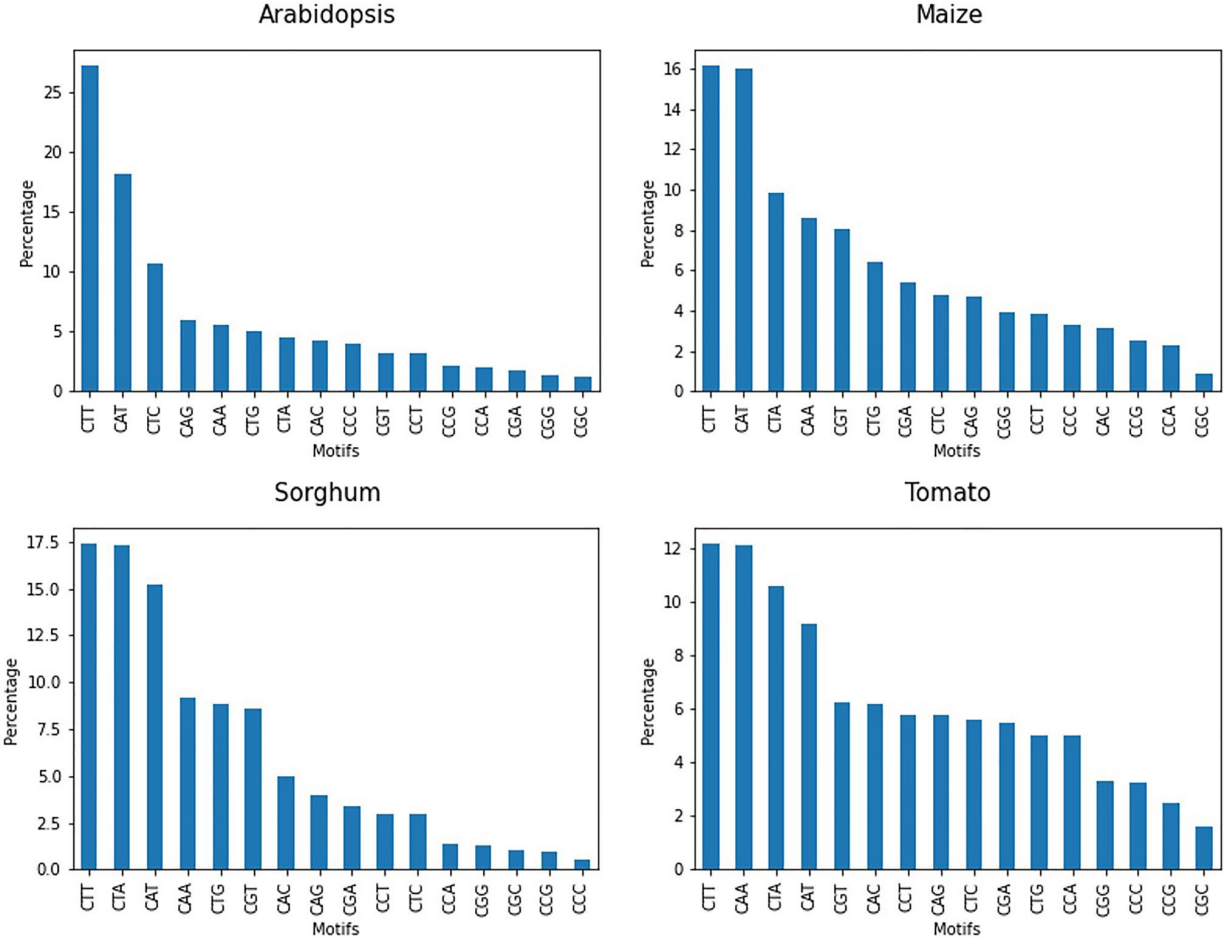
Proportion of the different methylated CHH motifs present in the genomes of *Arabidopisis*, maize, sorghum, tomato

At the genomic level, CHH methylation can be found in 16 different motifs, as the H nucleotide can be C, T, or A. To explore which motifs are most common in CHH methylation and more likely to influence recombination, a count of methylated CHH motifs in the genomes of the four species evaluated was performed. The results indicate that the CTT motif is the most abundant methylated motif in all species, followed by CAT, CTA, and CAA (Fig. 4). Similar results have been obtained by Gouil and Baulcombe (2016) who found that CHH methylation is dense at CAA and CTA in Arabidopsis and maize, at CAA and CAT in tomato, and at CTA in rice. They even suggest a detailed evaluation of the subcontexts within the CHG and CHH methylomes because many of them are binding sites and are involved in different cellular pathways.

On the other hand, evidence obtained by Horton et al. (2012) demonstrates the association between A-rich motifs and crossover frequency in Arabidopsis*.* Other authors such as (Choi et al. 2013) report that CTT and AAA motifs are associated with high recombination frequency in TSSs for the same species. They suggest that the overlap between these motifs, and the H2A.Z and LND regions may contribute to nucleosome positioning or chromatin organization at promoters, with consequences for recombination. For maize, (Kianian et al. 2018) found motifs with A/T content near to crossovers. More recently, (Rowan et al. 2019) reported that the poly(A) and CTT/GAA repeat motifs constituted approximately 90% of occurrences within the 500 bp region, nearly to cross-overs in Arabidopsis. Meanwhile, in tomato, (de Haas et al. 2017) report rich AT-rich DNA motifs at recombination prone regions, and (Rommel Fuentes et al. 2020) also report the finding of sequence motifs rich in cytosines, thymines, and adenines near crossover hotspots also in tomato.

Studies such as (Choi et al. 2018) report in Arabidopsis that SPO11-oligo hotspots that are involved in crossover events and lead to the formation of DNA double-strand breaks are rich in AT motifs. They propose that these exclude nucleosomes that are abundant in genes and transposons, reporting also that there are positive correlations between SPO11-1 binding sites and methylation in all contexts at chromosomal arms, and also negative correlations near the centromere. Based on the reports of these previous studies and the results presented in this paper, it is possible that methylation in CHH contexts, especially for motifs containing A or T nucleotides, may be involved in crossover recombination.

It should be noted that the results presented in this research are the product of a bioinformatics analysis between public data on recombination rates and methylomes, and are not the product of planned experiments. This could explain why correlations are weak in Arabidopsis and maize. It is also possible that these genomes have different relationships between their methylomes and chromosomal recombination, which were not captured. It will be necessary to explore other datasets and new methods to gain a better understanding within each species. The relative distribution of methylation domains varies by species: in maize, sorghum, and rice, CG and CHG methylation occurs in heterochromatic regions and is enriched in TEs and intergenic regions, but is reduced in genes. Meanwhile, rice has the highest CHH methylation, which is largely due to the RNA directed DNA methylation (RdDM) pathway (Vafadarshamasbi et al. 2022), which explains the findings of (Peñuela et al. 2022) who based their machine learning model just in this feature.

The datasets of all species were evaluated to choose the best machine-learning regression model using the Auto-Sklearn package. For all datasets, the Extra Trees model is chosen as the best model or is in the top positions of the recommended models. Based on this, the Extra Trees model is chosen to predict the recombination rates of the four species using the methylated cytosine counts from CG, CHG, and CHH contexts as features. The recommended parameters and feature transformations obtained by Auto-Sklearn were evaluated for each species. Since their increase in prediction performance is too small, the default model parameters and original features were maintained. Evaluations of this model using individual features or combinations of them for each species indicate that the best predictions are obtained with all three features as input. Extra Trees seems to be a powerful model with this type of data and approach as (Peñuela et al. 2022) also chose it to predict rice recombination. However, the Extra Trees model in rice trained only with methylation in the CHH context gives the best prediction results, while for the data evaluated in this study the best results are obtained using all three methylation contexts as input features.

The prediction results obtained using this setting are different for each species. For Arabidopsis, the coefficients of determination *R*^2^ are close to zero or negative for all five chromosomes, indicating that the model cannot reproduce the recombination rates using the methylation data. Correlation values for Arabidopsis chromosomes are greater than zero, even for chromosomes 1 and 5 they approach 0.5, providing evidence that the model can at least indicate recombination trends on those chromosomes (Table 1). Similar results are obtained for maize, where the predictions are far from the expected according to the *R*^2^ of the ten chromosomes, and only chromosomes 3, 5, 7, and 10 have correlation values superior to 0.5 (Table 2). It is to be expected that for Arabidopsis and maize the predictions are not good since the features used (counts of methylated cytosines in contexts CG, CHG, and CHH) do not show strong relationships with recombination rates (Figs. 1, 2, 3).

**Table 1:** Performance of chromosome recombination rates predictions of *Arabidopsis* using the Extra Trees model trained with CG, CHG, and CHH methylation data

Chromosome	*R*^2^	Correlation	*MSE*
1	0.14	0.44	0.02
2	− 0.42	0.14	0.02
3	− 0.13	0.25	0.03
4	− 0.43	0.11	0.05
5	0.23	0.49	0.01

**Table 2:** Performance of chromosome recombination rates predictions of maize using the Extra Trees model trained with CG, CHG, and CHH methylation data

Chromosome	*R*^2^	Correlation	*MSE*
1	0.13	0.39	0.004
2	0.04	0.49	0.004
3	0.27	0.54	0.005
4	0.17	0.43	0.005
5	0.24	0.54	0.003
6	0.16	0.42	0.004
7	0.31	0.58	0.006
8	0.11	0.34	0.006
9	− 0.06	0.18	0.015
10	0.27	0.57	0.008

**Fig. 5:**
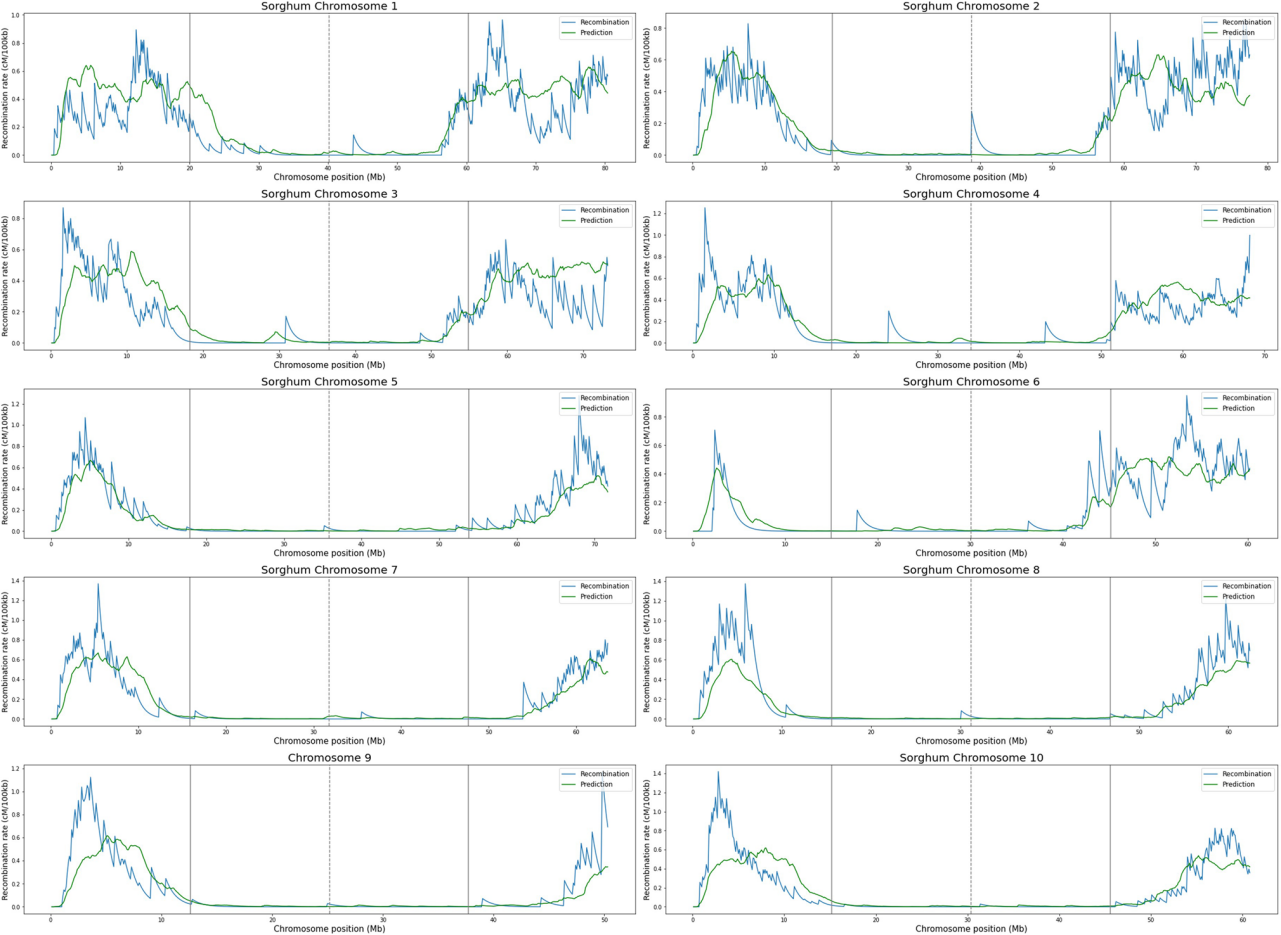
Predictions of recombination rates for sorghum chromosomes by the Extra Trees machine learning model using methylated cytosine counts in CG, CHG, and CHH contexts as features. For chromosome length, the continuous gray lines represent quartiles 1 and 3, while the dashed gray line represents quartile 2

On the other hand, sorghum predictions show a different pattern. The mean value of *R*^2^ for the 10 chromosomes is 0.65 ± 0.11. For some chromosomes, such as 5 and 7, the values rise to 0.79 and 0.77, respectively, showing that the model can adequately reproduce the recombination rates for this species. In the same way, correlation values between predictions and recombination rates are high with a mean value of 0.83 ± 0.06 (Table 3). These results indicate that the model performs well in predicting recombination rates for sorghum using methylated data as input. In contrast to Arabidopsis and maize, CG, CHG, and CHH features show clear negative and positive trends with recombination (Figs. 1, 2, 3), which helps to better train the model allowing better predictions. The predicted recombination landscapes on sorghum chromosomes can be seen in Fig. 5.

**Table 3:** Performance of chromosome recombination rates predictions of sorghum using the Extra Trees model trained with CG, CHG, and CHH methylation data

Chromosome	*R*^2^	Correlation	*MSE*
1	0.48	0.76	0.02
2	0.69	0.83	0.01
3	0.48	0.77	0.01
4	0.60	0.78	0.02
5	0.79	0.92	0.01
6	0.72	0.85	0.01
7	0.77	0.88	0.01
8	0.74	0.94	0.02
9	0.57	0.77	0.02
10	0.64	0.81	0.02

**Fig. 6:**
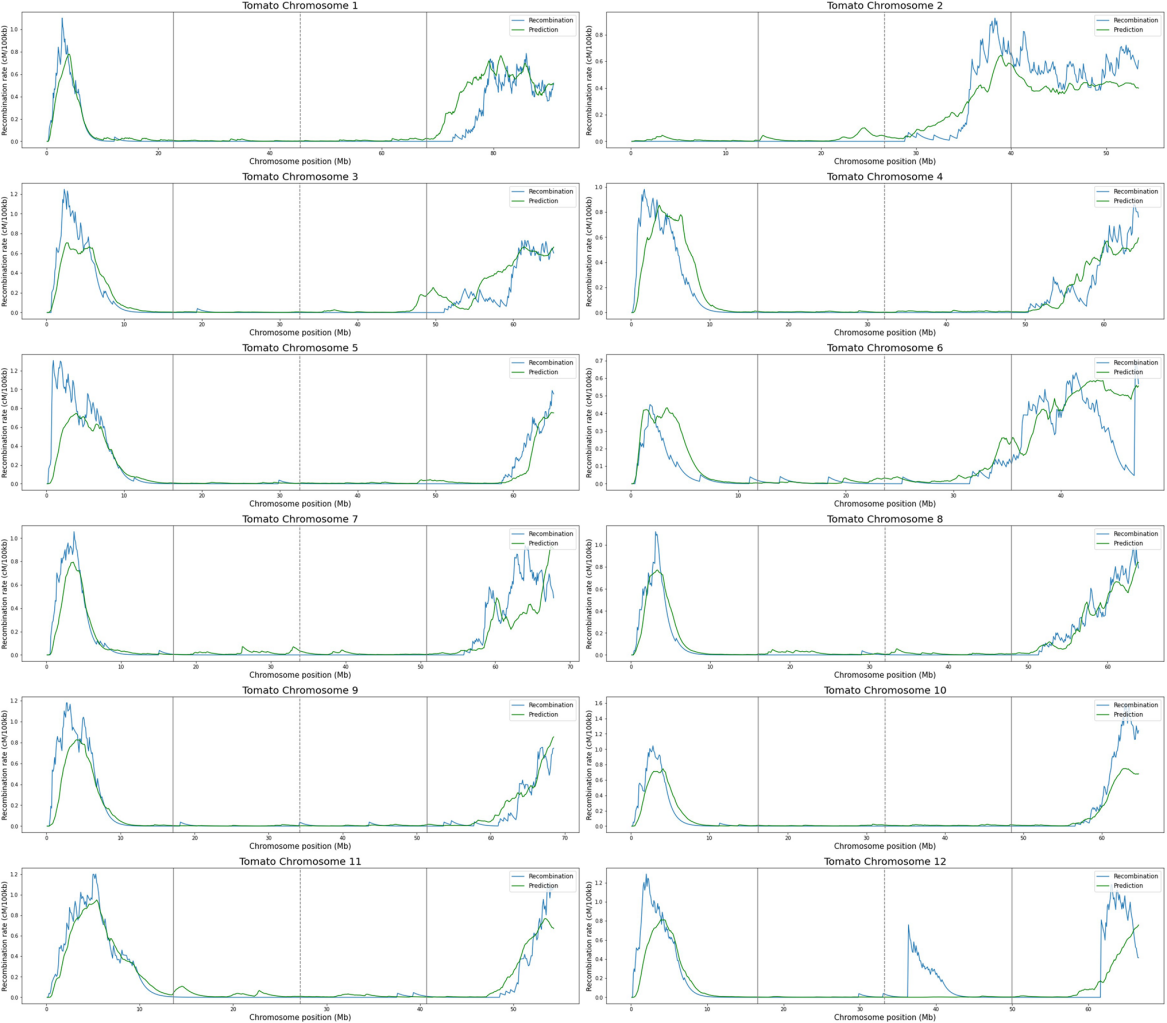
Predictions of recombination rates for tomato chromosomes by the Extra Trees machine learning model using methylated cytosine counts in CG, CHG, and CHH contexts as features. For chromosome length, the continuous gray lines represent quartiles 1 and 3, while the dashed gray line represents quartile 2

In the case of tomato, the results are better than those of sorghum. The mean value of *R*^2^ for the twelve chromosomes is 0.76 ± 0.05, with chromosomes 8 and 12 both reaching 0.92. The mean correlation value is 0.90 ± 0.05 (Table 4). The predicted recombination landscapes on tomato chromosomes can be seen in Fig. 6. These predictions in sorghum and tomato are even better than those obtained by Peñuela et al. (2022) in rice, who planned the experiments and obtained the methylomes and recombination rates of the same varieties. They report a mean coefficient of determination of 0.32 for the Azucena variety and 0.21 for the IR64 variety, and correlation values of 0.67 and 0.65, respectively.

**Table 4:** Performance of chromosome recombination rates predictions of tomato using the Extra Trees model trained with CG, CHG, and CHH methylation data

Chromosome	*R*^2^	Correlation	*MSE*
1	0.75	0.89	0.01
2	0.83	0.95	0.01
3	0.77	0.88	0.01
4	0.75	0.87	0.01
5	0.73	0.88	0.02
6	0.53	0.83	0.01
7	0.76	0.88	0.01
8	0.92	0.96	0.00
9	0.83	0.92	0.01
10	0.78	0.94	0.02
11	0.92	0.96	0.00
12	0.58	0.81	0.04

**Fig. 7:**
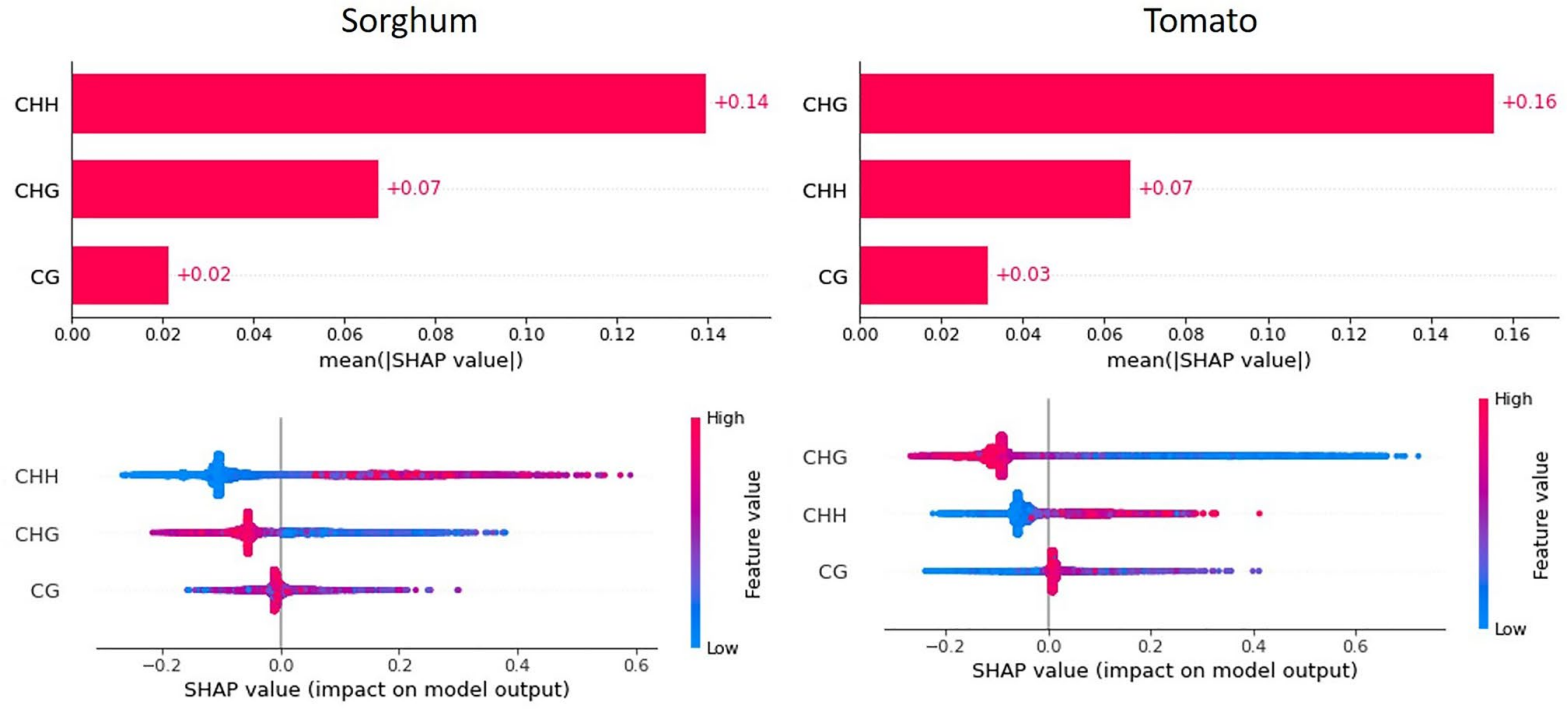
Shap values and contributions of features CG, CHG, and CHH to the prediction of recombination rates in sorghum and tomato. Shap values at the top and Shap summary graph at the bottom

For sorghum and tomato, the Extra Trees model is able to obtain good performance in predicting recombination rates, this is also evident in the low *MSE* values for the predictions of both species. However, the contribution of each feature to the model is not the same for sorghum and tomato. For the former, the CHH context has the largest contribution to model predictions, followed by CHG and CG, with the highest CHH values producing the greatest impact. For the latter, the CHG context has the largest contribution to the predictions, followed by CHH and CG, with the lowest CHG values having the greatest impact on the predictions (Fig. 7).

**Fig. 8:**
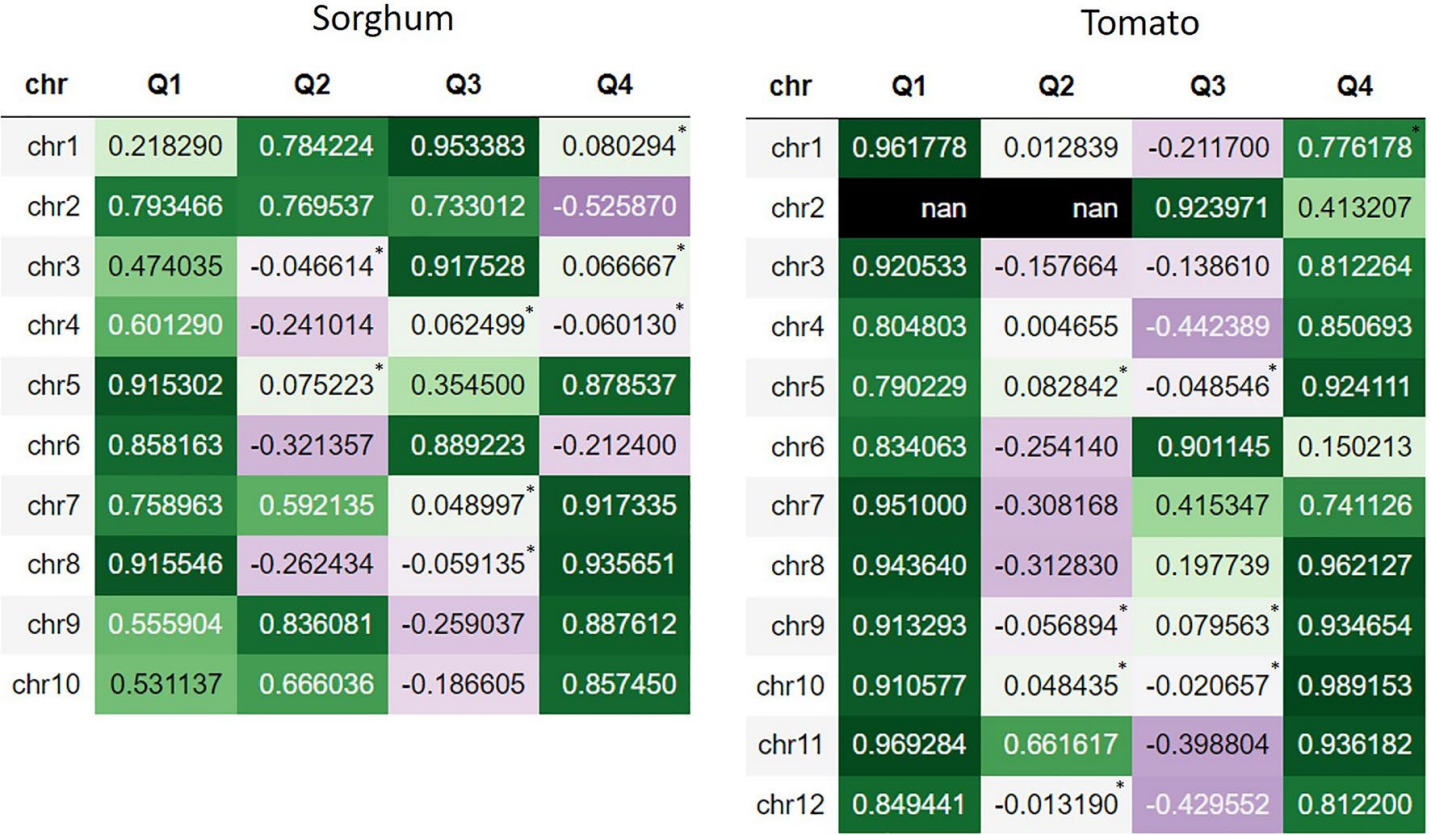
Pearson correlation values between the predicted recombination rates and the experimental recombination rates using the ExtraTrees model and the methylation data as input for all chromosomes of sorghum and tomato. In Q1, correlation values for the first quartile of the chromosome, Q2 for the second, Q3 the third, and Q4 the fourth. The purple color represents negative values and the green color represents positive values. The higher the correlation value, the higher the color intensity. Non-significant values are marked with a star

For these two species, the recombination landscape varies along the chromosomes showing higher recombination rates in the chromosome arms, with the predictions being able to reproduce these general patterns. However, the performance of the predictions is not the same on each section of the chromosome. To measure this, the chromosomes were divided into quartiles and the performance of the model was evaluated in each quartile (Fig. 8). For sorghum, the Q1 and Q4 quartiles, those containing the distal regions of the chromosome arms, show a higher correlation between predicted and experimental values compared to those of the Q2 and Q3 quartiles, which contain the inner arms of the chromosomes and the centromere region. In the distal quartiles, correlation values are higher because the predictions reproduce positive trends where recombination increases and negative trends where recombination decreases, drawing peaks.

In contrast, many inner quartiles show low or negative correlation values. This is because, for many windows, the experimental recombination rate is zero or near zero, and the model does not predict trends that can increase the correlation value. For this reason, the performance of the model is low. However, although the model does not perform well in the inner regions of chromosomes, it is able to indicate the absence of recombination, which is valuable for breeding, the final goal. Therefore, it is necessary to pay attention to how the data are analyzed; the landscape figures and the model performance are complementary.

In the inner quartiles of sorghum, where correlations between experimental and predicted rates were high, it was because the model replicates the trends of the onset of the recombination hill. In the case of tomato, a similar behavior is observed. The distal quartiles show higher model performance compared to the inner quartiles, the explanation being the same as for sorghum. However, in the case of tomato, the least inner quartiles show positive correlation values, which is due to the fact that the recombination peaks are further away from the inner region of the chromosome and appear almost entirely in the distal quartiles. For tomato’s chromosome two, the experimental recombination rates were zero in the windows of Q1 and Q2 because recombination is suppressed in this arm of the chromosome, and correlation could not be calculated with respect to the predicted values.

Both trained models seem good enough for their datasets, however, based on Shap values, the two Extra Trees models trained on sorghum and tomato differ in the contribution of the three features to the predictions, and the question remains whether both models would be good on other data sets. To evaluate this, a model trained on all sorghum chromosomes was used to predict recombination in tomato and the model trained on all tomato chromosomes to predict recombination in sorghum. The results show that the predictions in sorghum using the model trained on tomato have a mean *R*^2^ of 0.28 ± 0.60 for the 10 chromosomes, with minimum values of − 0.47 and − 1.02 for chromosomes 1 and 3, respectively, and maximum values of 0.74 and 0.78 for chromosomes 5 and 8, respectively. Meanwhile, correlation values have a mean of 0.81 ± 0.08, with minimum values of 0.66 and 0.70 for chromosomes 1 and 3, respectively, and maximum values of 0.90 and 0.91 for chromosomes 7 and 8, respectively (Table 5).

**Table 5:** Performance of chromosome recombination rates predictions of sorghum using the Extra Trees model trained with tomato methylation data

Chromosome	*R*^2^	Correlation	*MSE*
1	− 0.47	0.66	0.07
2	0.48	0.85	0.02
3	− 1.02	0.70	0.07
4	0.07	0.72	0.04
5	0.71	0.88	0.01
6	0.19	0.82	0.03
7	0.78	0.90	0.01
8	0.74	0.91	0.02
9	0.69	0.85	0.01
10	0.65	0.82	0.02

For tomato, the predictions of the model trained with sorghum data show a mean *R*^2^ of 0.65 ± 0.26 for the twelve chromosomes, with minimum values of 0.23 and 0.32 for chromosomes 3 and 4, respectively, and maximum values of 0.88 for chromosomes 8, 9, and 10, and 0.90 for Chromosome 11. In addition, correlation values have a mean of 0.83 ± 0.12, with minimum values of 0.63 for Chromosome 3 and 0.67 for chromosomes 4 and 5, and maximum values of 0.94 for chromosomes 8 and 9, and 0.95 for chromosomes 10 and 11 (Table 6).

**Table 6:** Performance of chromosome recombination rates predictions of tomato using the Extra Trees model trained with sorghum methylation data

Chromosome	*R*^2^	Correlation	*MSE*
1	0.33	0.68	0.03
2	0.62	0.84	0.02
3	0.23	0.63	0.05
4	0.32	0.67	0.04
5	0.34	0.67	0.06
6	0.78	0.88	0.00
7	0.87	0.93	0.00
8	0.88	0.94	0.00
9	0.88	0.94	0.00
10	0.88	0.95	0.01
11	0.90	0.95	0.00
12	0.79	0.89	0.02

These predictions are interesting considering that the models are being trained on genetically distant plant methylomes. In fact, sorghum and tomato belong taxonomically to different classes, indicating that they are not closely related, sorghum being a monocotyledonous plant and tomato a dicotyledonous one. It is surprising how these methylation data can be used to predict recombination even when training models on such distant species. For both cases, the average performance in *R*^2^ and correlation of predictions in their own datasets exceed the results obtained by Peñuela et al. (2022) in rice, demonstrating the broad potential of methylomes in crop improvement. For example, the model trained on the sorghum methylome could be useful to shed light on sugarcane recombination, which is a species very close to sorghum, but with high genomic complexity product of hybridization events and high ploidy. For instance, the estimation of recombination rates in sugarcane is not frequent because hybrid clones are generally grown in the field.

Further work is needed to clarify the influence of the methylome on chromosomal recombination in plants, and more experimental data will be needed to test this methodology and create a solid background in this field. Using these datasets, good predictions are obtained for sorghum and tomato, but not so good for Arabidopsis and maize. This is probably because the methylomes and genetic maps used here are not compatible or because other methodologies will be needed to extract information from the data to obtain better predictions. The door remains open in this new field of research for the community to explore methylomes in other plant species. Also, the data and the code presented in this work can be used to propose improvements or new methods of recombination prediction (https://github.com/maurope/MethylRec).

## Conclusion

This study reported that methylation in the CHH context was positively associated with chromosomal recombination rates in Arabidopsis, maize, sorghum, and tomato, having a similar pattern recently found in rice. This indicates that such a pattern is common across different plant species and suggests a possible role of CHH methylation in chromosomal recombination. In the context of CHH methylation, the prevalence of methylated cytosines showed a clear pattern, with the CTT motif being the most abundant among all the species evaluated. Additionally, the CAT, CTA, and CAA motifs were also frequently observed, further highlighting their relevance in the methylation landscape across these species. With respect to methylation in CG and CHG contexts, the results showed high levels of methylation in mid-chromosomal regions close to centromeres, which is in agreement with what is widely reported in plants. However, correlation values between cytosines methylated in different contexts and recombination rates varied among the four species evaluated, being mild for Arabidopsis and maize, and strong for sorghum and tomato. According to these results, the count of methylated cytosines in all contexts were evaluated as features to train machine learning models for predicting recombination rates in the focused species. The results showed that the Extra Trees model was the best for predicting recombination rates from methylation data and that the default parameters are sufficient for good performance. Predictions were especially good for sorghum and tomato datasets, showing the significant potential of the proposed methodology to make predictions on chromosomal recombination at the 100 Kb scale.

## Data Availability

Enquiries about data availability should be directed to the authors.
